# The epigenetic landscape of heterosis: integrating multi-layer regulatory insights and navigating the path to causality

**DOI:** 10.3389/fpls.2026.1838658

**Published:** 2026-05-19

**Authors:** Putri Wijayanti, Yuko Wada, Toshiro Ito

**Affiliations:** Division of Biological Science, Graduate School of Science and Technology, Nara Institute of Science and Technology, Ikoma, Nara, Japan

**Keywords:** chromatin, DNA methylation, epigenetics, heterosis, histone modification, plant breeding, small RNAs

## Abstract

In some crosses, the hybrid progeny perform better than their parental inbred lines in terms of beneficial traits (e.g., greater biomass, faster development), a phenomenon known as hybrid vigor or heterosis. While classical genetic paradigms explain much of its basis, how these interactions translate into specific molecular frameworks remains a significant challenge. Mounting evidence indicates that epigenetic regulation—encompassing chromatin states, histone modifications, DNA methylation, and small RNAs—reshapes the hybrid transcriptional landscape. While many studies provide compelling correlations between epigenetic remodeling and heterotic phenotypes, a definitive mechanistic synthesis remains elusive due to the complexity of distinguishing cause from consequence. This review integrates current knowledge of these multilayered regulatory systems, emphasizing the redundant and hierarchical nature of epigenetic interactions that sustain the heterotic state. We highlight the transition from descriptive profiling to functional validation, and discuss how emerging tools such as targeted epigenome editing for recreating optimal hybrid methylotypes may finally bridge the gap between epigenetic variation and hybrid vigor.

## 1 Introduction

Plant productivity is fundamental to meeting the demands of a growing global population. Heterosis, wherein hybrid progeny outperform their parental inbred lines, has been central to improving yield in major crop species such as maize (*Zea mays*), wheat (*Triticum aestivum*), and rice (*Oryza sativa*). Enhanced hybrid performance is defined as performance exceeding either the parental mean or the performance of the superior parent (Springer and Stupar, 2007). Despite its significant influence on agriculture, the molecular mechanisms that underlie heterosis have not yet been unified into a coherent mechanistic framework.

The classical genetic paradigm hypothesizes that heterosis occurs through three types of allelic interactions: dominance, overdominance, and epistasis (Springer and Stupar, 2007; Paril et al., 2024). Dominance involves complementation by a superior allele; overdominance results from interactions between alleles from the two parents that confer enhanced function; and epistasis reflects interactions among alleles across multiple loci. However, how these allelic interactions are coordinated within gene regulatory networks and produce heterotic phenotypes remains incompletely understood.

Gene expression variation serves as a key mechanistic bridge between allele variation and phenotypic diversity (Tsouris et al., 2024). In a hybrid, a gene can exist in either homozygous or heterozygous states, and homozygous genes may still show differential expression between the hybrid and its parental lines (Botet and Keurentjes, 2020). Recent reports comparing gene expression between hybrid and parental lines that show either allele-specific expression or a degree of non-additive gene expression (Zhou et al., 2019; Zhang J. et al., 2025; Pitz et al., 2025a, 2025b) have identified altered gene expression in the hybrids. Although it has been difficult to establish a common causal relationship between the massive gene expression changes in hybrids and their heterotic phenotypes, gene expression regulation must play a role.

**Figure 1:**
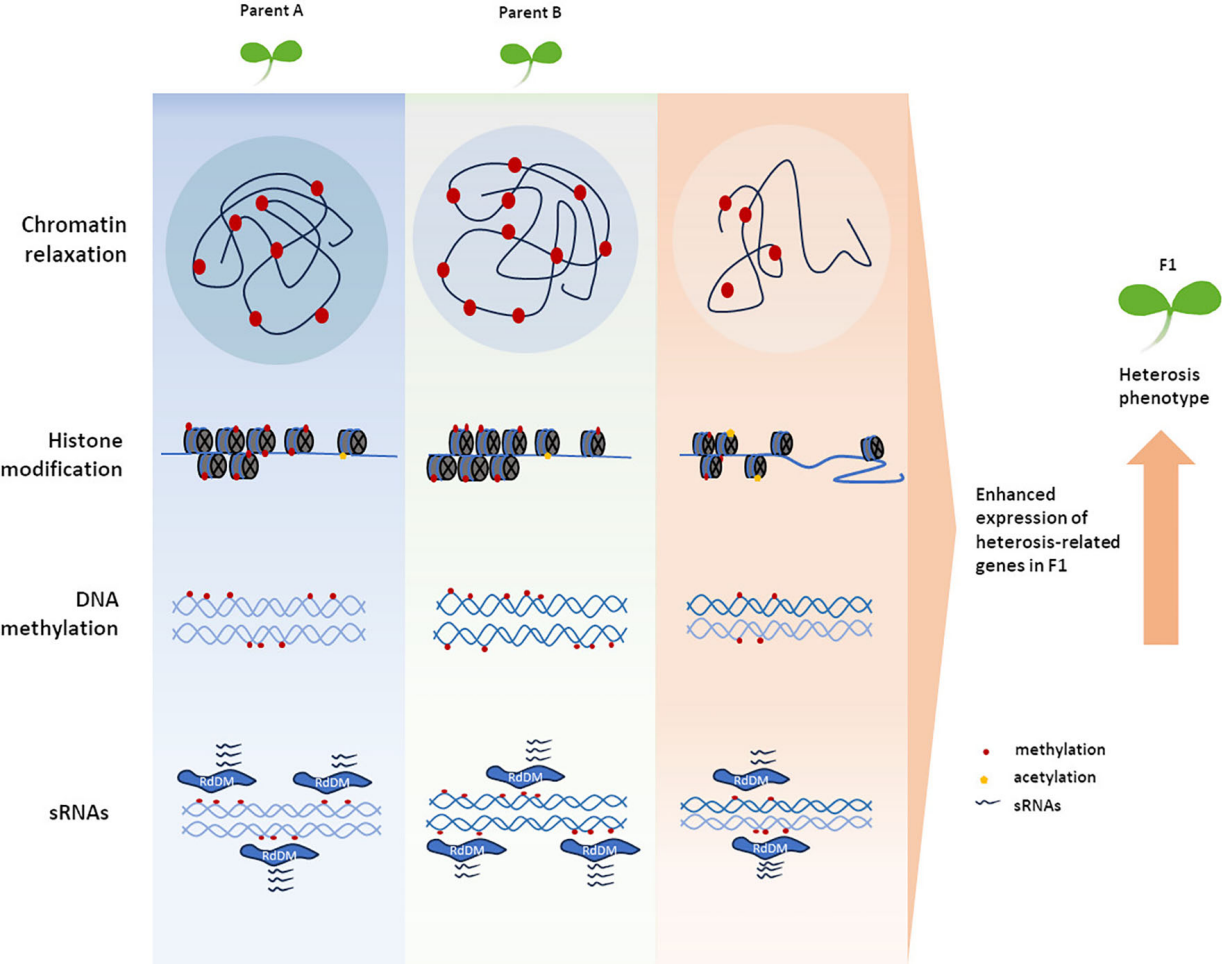
Epigenetic regulation contributes to heterosis. Compared to their two parental lines, F1 hybrids often exhibit chromatin relaxation and altered histone modification patterns that enhance chromatin accessibility and interactions between loci. Changes also occur in DNA methylation, with hybrids generally showing altered methylation levels relative to their parents, together with both inherited and newly established methylation patterns. Small RNAs (sRNAs) mediate these methylation dynamics, for example through the RNA-directed DNA methylation (RdDM) pathway, helping maintain epigenetic homeostasis and regulate gene expression. This multilayered epigenetic regulation contributes to the transcriptional reprogramming of heterosis-related genes and may underlie the manifestation of heterosis in plants.

Epigenetic regulation involves heritable yet reversible mechanisms such as chromatin reorganization, histone modification, DNA methylation, and noncoding RNA activity, all of which affect gene expression (Bird, 2007). Epigenetics may therefore add further explanation beyond current classic genetic theory and reveal the complexity of gene expression in hybrids (**Figure 1**). Although experimental manipulation of epialleles to directly demonstrate the role of epigenetic regulation in heterosis remains limited, recent studies have shown that in intraspecific hybrids of *Arabidopsis thaliana* derived from nearly isogenic parents differing in their epigenetic states, epigenetic variation influences gene expression and can give rise to heterotic phenotypes (Lauss et al., 2018; Kakoulidou et al., 2024). In interspecific hybrids and polyploid plants, which have higher allele variation, heterosis also seems to be related to changes in epigenetic markers (Chen et al., 2025; Peng et al., 2025; Miao et al., 2024; Quan et al., 2025). This compelling evidence suggests that epigenetic regulation may be involved in allelic interaction that affects gene expression regulation, therefore contributing to heterosis. Here, we review current knowledge on the contribution of epigenetic remodeling to heterosis.

## 2 Global chromatin reorganization: a structural blueprint for hybrid vigor

In eukaryotic genomes, chromatin organization is shaped by a coordinated regulatory network that involves most aspects of epigenetic regulation; the resulting chromatin landscape directly affects chromatin accessibility, which in turn modulates transcription (Huang et al., 2025). Across diverse plant hybrid systems, recent studies have shown that hybridization can induce substantial reorganization of chromatin architecture, resulting in genome configurations in both intraspecific and interspecific hybrids that are not simply additive relative to their parental lines (Hu et al., 2022; Wang et al., 2022; Jiao et al., 2024; Gao et al., 2024; Liu et al., 2025).

In maize reciprocal hybrids, hybrid-specific long-range chromatin interaction, including novel inter-allelic contacts absent in either parent, emerges and is associated with non-additive gene expression (Liu et al., 2025). Similarly, *Arabidopsis* hybrids exhibit increased frequencies of long-range chromatin interactions, together with increased crosstalk between euchromatin and heterochromatin, indicating a global reorganization of genome architecture in the hybrid state (Gao et al., 2024). However, despite these consistent observations, studies to date of genome architecture in hybrids remain largely descriptive at the genome-wide level. Further studies establishing direct causal links between defined chromatin structural changes, the regulation of specific genes, and the consequent heterotic phenotypes will be essential for exploring the role of chromatin changes in intraspecific hybrid heterosis.

*Brassica napus* hybrids also display A/B genomic compartment switches that coincide with increased chromatin accessibility and genes exhibiting expression level dominance, supporting enhanced transcriptional plasticity in superior heterotic hybrids (Hu et al., 2022). Similarly, in tetraploid wheat, quantitative trait loci (QTLs) associated with yield and plant architecture are enriched within differentially-interacting chromatin loops, where long-range contacts link these QTLs to genes whose expression correlates with the corresponding phenotypic traits, suggesting a potential regulatory role for chromatin interactions (Jiao et al., 2024). However, in interspecific hybrids and polyploid plants, such chromatin and transcriptional changes cannot be fully disentangled from the effects of genome duplication and sequence divergence, both of which may independently influence gene expression.

## 3 Histone modification dynamics: fine-tuning the hybrid transcriptional rhythm

Histone modifications, including methylation, acetylation, ubiquitination, and phosphorylation, occur primarily at lysine and arginine residues in the N-terminal tails of histones H2A, H2B, H3, H4, and H1 (Luger et al., 1997; Nakamura et al., 2025). These modifications may promote an open chromatin state that facilitates gene expression or create a repressive chromatin environment that inhibits transcription (Zhang et al., 2015). Histone modifications are deposited by specific enzymes, such as histone acetyltransferases, deacetylases, methyltransferases, and demethylases (Bannister and Kouzarides, 2011). In *Arabidopsis*, hybrids with reduced activity of the key methyltransferase responsible for the deposition of the repressive histone 3 lysine 27 trimethylation (H3K27me3) mark exhibit a diminished heterotic phenotype, attributed to a shorter range of chromatin interactions (Gao et al., 2024). Continued investigation of the histone modification machinery will be crucial for determining how these enzymes collectively orchestrate transcriptional reprogramming to produce heterotic phenotypes.

Although some genome-wide profiling studies of histone modifications have shown less histone modification in hybrids compared to their parents (He et al., 2010; Zhu et al., 2017; Ma et al., 2023), others have reported a connection between histone modifications and hybrid phenotypes (Ni et al., 2009; Yang et al., 2021). In both diploid and allotetraploid *Arabidopsis* hybrids, histone-mediated regulation of circadian clock genes, particularly *CIRCADIAN CLOCK ASSOCIATED 1* (*CCA1*) and *LATE ELONGATED HYPOCOTYL* (*LHY*), appears to modulate transcriptional rhythms that control biomass accumulation (Ni et al., 2009). Heterosis is also observed in bacterial defense, with increased H3K9ac levels at the *CCA1* promoter acting as an epigenetic priming mark that induces a robust pre-dawn burst of *CCA1* expression (Yang et al., 2021). These findings suggest that histone modification contributes to fine-tuning central regulatory genes, although direct causal links between specific histone modifications and heterotic phenotypes remain to be identified.

## 4 DNA methylation: a flexible modulator of subgenome harmony

Cytosine methylation in the CG, CHG, and CHH contexts is a key epigenetic mark that modulates gene silencing, genome stability, and transcriptional regulation. Specific biochemical pathways maintain each context: CG methylation (mCG) by DNA METHYLTRANSFERSE 1 (MET1), CHG methylation (mCHG) by CHROMOMETHYLASE 3 (CMT3), and CHH methylation (mCHH) by CHROMOMETHYLASE 2 (CMT2) or DOMAINS REARRANGED METHYLTRANSFERASE 2 (DRM2) (Law and Jacobsen, 2010; Wada et al., 2003). Promoter methylation typically represses gene expression (Lang et al., 2017), whereas gene-body methylation is often associated with active transcription and may aid stress-induced gene activation (Lee et al., 2023).

Recent studies have demonstrated that locus-specific variation in DNA methylation between isogenic parental lines of *Arabidopsis* hybrids can induce heterosis that affects gene expression (Lauss et al., 2018; Kakoulidou et al., 2024). Furthermore, studies using epigenetic recombinant inbred lines (epiRILs) have demonstrated that altered DNA methylation patterns alone, even in the absence of significant genetic variation, can contribute to heterotic phenotypes (Dapp et al., 2015). Consistent with this, the *ddm1* mutation in *Arabidopsis* hybrids has been suggested to cause stochastic changes in DNA methylation that contribute directly or indirectly to heterosis, where *DDM1*-mediated regulation maintains the gene expression balance required for hybrid vigor (Kawanabe et al., 2016; Matsuo et al., 2025).

DNA methylation in hybrids and polyploids is remodeled in a locus- and context-specific manner, often correlating negatively with gene expression (Kakoulidou et al., 2024; Zhang X. et al., 2025; Shan et al., 2024). In radish hybrids, methylation changes in promoters and trait-relevant genes modulate heterosis-related traits (Zhang X. et al., 2025). Meanwhile, in some polyploid plants like wheat, new epigenetic configurations can harmonize subgenomes and influence the divergent expression of homoeologs (Miao et al., 2024; Shan et al., 2024). Collectively, these studies highlight that DNA methylation acts as a flexible epigenetic modulator influencing expression and phenotype in hybrids.

## 5 Small RNA-mediated regulation: silencing and stability in the hybrid state

**Figure 2:**
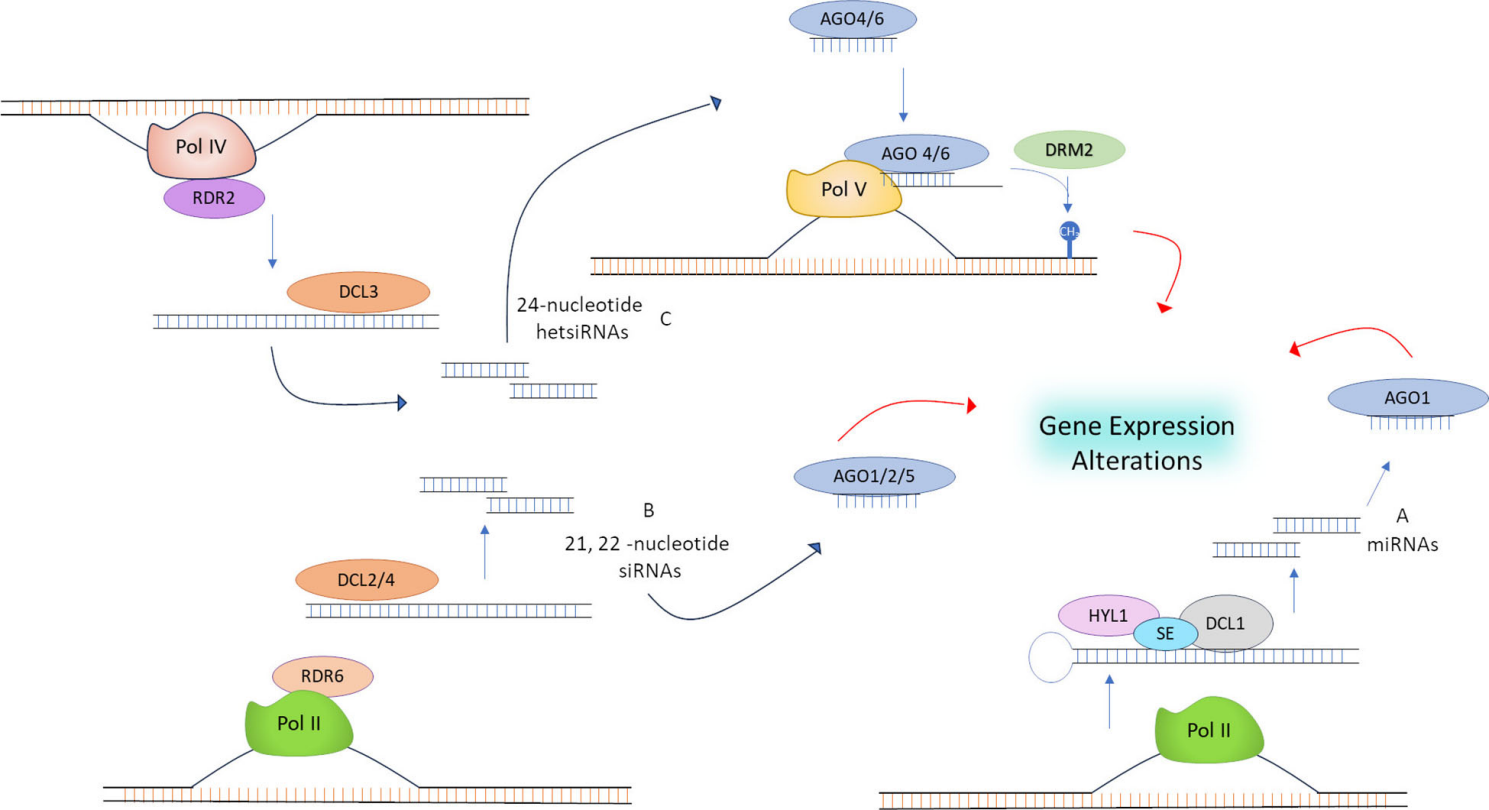
Potential small RNA-mediated regulation of gene expression and its contribution to gene expression regulation in a hybrid. miRNAs are primarily transcribed from *MIR* genes by RNA polymerase II (Pol II) and processed in the nucleus by DICER-LIKE 1 (DCL1) together with HYPONASTIC LEAVES 1 (HYL1) and SERRATE (SE). **(a)** Mature miRNAs are predominantly loaded into ARGONAUTE 1 (AGO1) and regulate gene expression post-transcriptionally through mRNA cleavage or translational repression, or by triggering secondary phased siRNA (phasiRNA) biogenesis. **(b)** Secondary siRNAs are generated from Pol II-derived transcripts through the activity of RNA-DEPENDENT RNA POLYMERASE 6 (RDR6) and processed by DCL2 and DCL4. These siRNAs are typically associated with AGO proteins and mediate post-transcriptional gene silencing, particularly in developmental regulation and stress response. **(c)** Heterochromatic siRNAs (hetsiRNAs) are mainly produced via Pol IV-dependent transcription, followed by RDR2-mediated dsRNA synthesis and DCL3 processing into 24-nt siRNAs. These hetsiRNAs associate with AGO4 or AGO6 and guide RNA-directed DNA methylation (RdDM) at Pol V-transcribed loci through the action of DOMAIN REARRANGED METHYLTRANSFERASE 2 (DRM2), leading to transcriptional silencing of transposable elements.

Endogenous small RNAs (sRNAs) in plants are categorized into three major classes: microRNAs (miRNAs), secondary small interfering RNAs (siRNAs), and heterochromatic siRNAs (hetsiRNAs) (Borges and Martienssen, 2015; Zhan and Meyers, 2023; Matzke and Mosher, 2014). miRNAs predominantly mediate post-transcriptional regulation through mRNA cleavage or translational inhibition (Zhan and Meyers, 2023). Secondary siRNAs can block transcription in cis and in trans, while hetsiRNAs serve as guides for *de novo* DNA methyltransferases in the RNA-directed DNA methylation (RdDM) pathway (Matzke and Mosher, 2014; Feng et al., 2010). sRNA-mediated regulation can modulate gene expression in hybrids and may thereby contribute to the manifestation of heterosis (**Figure 2**).

Hybrids often display sRNA profiles that are distinct from those of their parental lines (Ha et al., 2009; Groszmann et al., 2011; Zhang et al., 2023; Hamid et al., 2023). Some reports indicate that these changes coincide with localized DNA methylation remodeling, suggesting that RdDM shapes the hybrid epigenetic landscape (Chodavarapu et al., 2012; Seifert et al., 2018). In maize hybrids, non-additively expressed miRNA and siRNA clusters affect plant height by targeting growth-related genes (Zhang et al., 2023). Similarly, in allotetraploid cotton, 24-nt siRNAs reinforce transposon silencing to stabilize the hybrid genome (Hamid et al., 2023). Nevertheless, heterotic phenotypes are still observed in the *nrpd1 nrpe1* mutant, which lacks key RNA polymerases for RdDM (Zhang et al., 2016). Similarly, a maize hybrid lacking *mop1* is also reported to retain heterosis, despite a substantial reduction in global 24-nt siRNA levels and changes in chromosome organization (Barber et al., 2012; Nobuta et al., 2008; Hasan et al., 2025). While small RNAs and the RdDM pathway are central to *de novo* DNA methylation in hybrids, the observation that heterosis persists even in mutants lacking key components of the RdDM machinery—such as the *nrpd1 nrpe1* double mutant in *Arabidopsis* (Zhang et al., 2016) and the *mop1* mutant in maize (Barber et al., 2012; Nobuta et al., 2008; Hasan et al., 2025)—indicates that siRNA biogenesis is not the sole driver of hybrid vigor. This suggests a hierarchical or redundant epigenetic buffering system, where other mechanisms—such as histone modification or MET1-mediated maintenance of DNA methylation—can sustain the heterotic state in the absence of the major siRNA pool. Thus, heterosis should be viewed as a multilayered integration of various epigenetic marks rather than a result of a single pathway.

## 6 Beyond correlation: navigating the path to functional causality

A major conceptual hurdle in hybrid epigenomics is the “chicken-and-egg” problem: determining whether epigenetic remodeling is a driver of heterosis or a downstream consequence of the hybrid state. Despite the wealth of descriptive “ome” data, the field remains largely at the stage of correlative observation. To transition to a mechanistic framework, the following strategies are essential:

### 6.1 Precision epigenome editing for functional testing

The development of CRISPR/Cas9-based tools offers a promising avenue. Traditional genetic mutants cause global disruptions, but emerging epigenome editing systems allow for targeted manipulation of epigenetic marks at specific loci. For example, the dCas9–SunTag system has been successfully adapted in *Arabidopsis* to induce targeted DNA methylation and activate silenced genes like *FLOWERING WAGENINGEN* (*FWA*) (Papikian et al., 2019). Similarly, the MS2-CRISPR/dCas9 system, utilizing histone modifiers like p300 and KRYPTONITE (KYP), has demonstrated the ability to modulate gene expression through precise histone modifications (Lee et al., 2019). Furthermore, CRISPR activation (CRISPRa) systems targeting key regulatory genes such as *AREB1* have improved complex traits like drought tolerance (Paixão et al., 2019). Applying these selective tools to “heterosis-related” genes can directly test whether specific epigenetic states are sufficient to induce heterotic phenotypes. Building upon these validated systems—such as the dCas9–SunTag-mediated methylation (Papikian et al., 2019) or histone-modifying MS2-CRISPR/dCas9 tools (Lee et al., 2019)—we propose a framework for ‘Synthetic Heterosis’ via targeted epigenome editing. Specifically, by employing these tools to recreate the optimal ‘methylotype’ of high-performing F1 hybrids directly in elite inbred lines, researchers could potentially bypass the biological constraints of traditional cross-breeding, such as genetic recombination or hybrid sterility. This strategy aims to artificially induce the 3D chromatin architecture and allelic expression balances that define heterotic vigor, extending the functional applications of dCas9-based modulation (Paixão et al., 2019) from single-trait improvement to the holistic engineering of hybrid vigor.

### 6.2 Decoupling genetic and epigenetic variation

To disentangle these factors, the use of Epigenetic Recombinant Inbred Lines (EpiRILs) is paramount. Studies in *Arabidopsis* have demonstrated that epigenetic variation alone can generate heterosis (Lauss et al., 2018). Expanding these designs to major crops—by crossing isogenic lines that differ only in specific chromatin landscapes—will allow us to quantify the “epigenetic component” of hybrid vigor. Integrating single-cell epigenomics with these backgrounds will further resolve the spatial and temporal windows critical for heterosis.

## 7 Conclusion: future perspectives on epigenome engineering for predictive breeding

Recent advances in heterosis research have provided insight into the molecular landscape underlying hybrid vigor. The integration of classical heterosis studies with epigenomic analyses suggests that uncovering specific regulatory mechanisms may yield reliable epigenomic markers for crop improvement.

Selective control of gene expression through epigenetic engineering—as demonstrated by the targeted activation of *FWA* via the dCas9–SunTag system (Papikian et al., 2019), the modulation of FLOWERING LOCUS T (*FT*) through histone modifiers (Lee et al., 2019), and the enhancement of stress tolerance via CRISPRa-targeted *ABA-RESPONSIVE ELEMENT BINDING PROTEIN 1* (*AREB1*) (Paixão et al., 2019)—provides new strategies for agricultural productivity. These tools will be essential not only for establishing causality in heterosis but also for developing the next generation of climate-resilient, high-yielding sustainable crops.
